# Identification and Validation of Candidate Genes Involved in Fatty Acid Content in Oil Palm by Genome-Wide Association Analysis

**DOI:** 10.3389/fpls.2019.01263

**Published:** 2019-10-15

**Authors:** Wei Xia, Tingting Luo, Yajing Dou, Wei Zhang, Annaliese S. Mason, Dongyi Huang, Xiaolong Huang, Wenqi Tang, Jihua Wang, Chunyu Zhang, Yong Xiao

**Affiliations:** ^1^Institute of Tropical Agriculture and Forestry, Hainan University, Haikou, China; ^2^Coconut Research Institute, Chinese Academy of Tropical Agricultural sciences, Wenchang, China; ^3^National Research Center of Rapeseed Engineering and Technology and College of Plant Science and Technology, Huazhong Agricultural University, Wuhan, China; ^4^Department of Plant Breeding, IFZ Research Centre for Biosystems, Land Use and Nutrition, Justus Liebig University Giessen, Giessen, Germany; ^5^Guangdong Key Laboratory for Crops Genetic Genetic Improvement, Crops Research Institute, Guangdong Academy of Agricultural Sciences, Guangdong, China

**Keywords:** *Elaeis guineensis*, FatB, fatty acid, genome-wide association analysis, palmitic acid

## Abstract

Oil palm (*Elaeis guineensis*) is the highest yielding oil crop per unit area worldwide, but its oil is considered unhealthy for human consumption due to its high palmitic acid content (C16:0). In order to facilitate breeding for fatty acid content in oil palm, genome-wide association analysis (GWAS) was used to identify and validate single-nucleotide polymorphism (SNP) markers and underlying candidate genes associated with fatty acid content in a diversity panel of 200 oil palm individuals. A total of 1,261,501 SNP markers previously developed using SLAF-seq (specific locus amplified fragment sequencing) were used for GWAS. Based on this analysis, 62 SNP markers were significantly associated with fatty acid composition, and 223 candidate genes were identified in the flanking regions of these SNPs. We found one gene (acyl-ACP thioesterase B genes) that was involved in fatty acid biosynthesis and that was associated with high palmitic acid content in the mesocarp. Over-expression of this gene caused a significant increase in palmitic acid content. Our study provides key loci that can be used for breeding oil palm cultivars with low palmitic acid content.

## Introduction

Oil palm (*Elaeis guineensis*, 2n = 32) is an important tropical oil crop. Among all oil crops, oil palm has the highest oil yield per unit area: its oil yield is more than 6 times that of peanut, 8 times that of soybean, and 10 times that of rapeseed (Huang, 2017). The world production of palm oil in 2017 was approximately 65 million tonnes (http://faostat3.fao.org/home/E). The two oil storage tissues of oil palm are the mesocarp and kernel, each of which produces oil with a different fatty acid composition. Palm oil usually refers to the oil extracted from the oil palm mesocarp, where palmitic acid (16:0) is the major fatty acid (50%). By contrast, in kernel oil, lauric acid (12:0) is the major fatty acid (50%). A principal objective in oil palm breeding is to decrease the palmitic acid content (16:0) and to increase the oleic acid (18:1) and linoleic acid content (18:2) in the oil product. Recently, by using conventional breeding methods, researchers in Malaysia have developed an oil palm variety with high oleic acid (approximately 52%), although this oleic acid content is still far lower than that in peanut (>75% in high oleic acid varieties) or rapeseed (>80%) (Rajanaidu et al., 2017). Improving fatty acid composition via conventional breeding, however, is a slow process in oil palm because of the plant’s long life cycle. The identification of genes controlling oil composition and the subsequent production of molecular markers linked to low palmitic acid content would be extremely helpful in accelerating the breeding and selection of oil palm.

**Figure 1:**
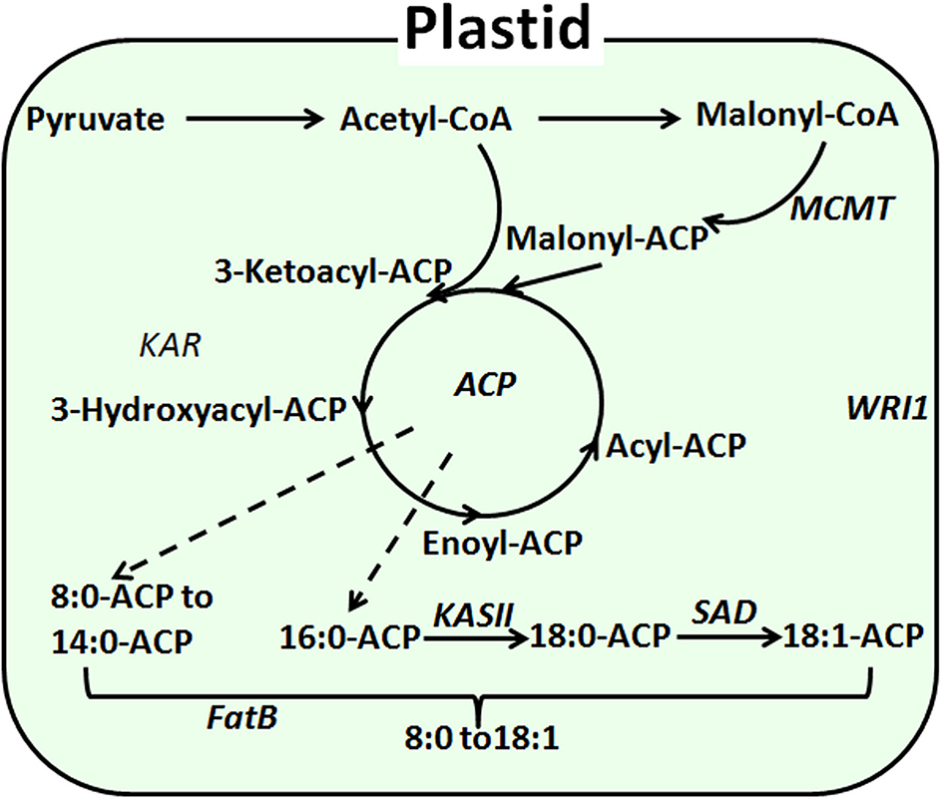
Diagram of fatty acid biosynthesis.

In *Arabidopsis*, substantial research has been done to elucidate genes involved in the fatty acid biosynthesis pathway, which occurs mainly in the plastids and endoplasmic reticulum. Initially, acetyl CoA carboxylase (ACCase) catalyzes the conversion of the precursor converacetyl-CoA into malonyl-CoA (Sasaki et al., 1997). Catalyzed by ketoly-ACP synthase (KAS), malonyl-CoA is subsequently polymerized at a frequency of two carbons per cycle into the acyl carbon chain and is combined with acyl carrier protein (ACP) (Choi et al., 2000). Finally, the elongation of the carbon chain is terminated as catalyzed by acyl-CoA thioesterase (FAT) (Hunt et al., 2002). Previous research has shown that FAT exists as FATA and FATB (Salas and Ohlrogge, 2002). FATA was thought to be able to bring about the termination of C18:0-ACP and C18:1-ACP (Mekhedov et al., 2002). FATB, however, may also be involved in the termination of saturated fatty acyl-ACP (Bonaventure et al., 2004). In *Arabidopsis thaliana*, overexpression of the *AtFatB1* gene can significantly increase palmitic acid content. The disruption of FatB expression resulted in a 56% reduction in palmitic acid and a 50% reduction of stearate in *Arabidopsis* seeds (Wilson et al., 2001; Buhr et al., 2002). Moreover, enhanced expression of FatB from *Umbellularia californica* was observed to significantly increase the lauric acid content (C12:0) in *Brassica napus* (Voelker et al., 1996) (**Figure 1**).

Association mapping is an efficient method for identifying molecular markers associated with agronomic traits based on natural populations with or without pedigree relationships (Flint-Garcia et al., 2003). The resolution of an association study depends on the extent and structure of linkage disequilibrium (LD) in the selected population. With the development of sequencing technology and high-throughput single-nucleotide polymorphism (SNP) markers, association mapping has been widely applied in different crops to dissect complex agronomic traits (Atwell et al., 2010; Huang et al., 2011; Li et al., 2013). However, there is still the problem of how to reduce the detection of spurious associations between traits and markers resulting from population structure. One solution is to perform association analysis between allelic and phenotype variation in a less structured population (Pritchard et al., 2000). Another solution for structured populations is to apply a mixed-model approach, which can decrease spurious associations (Sorkheh et al., 2008). Compared with QTL (quantitative trait loci) mapping in biparental cross populations, association analysis using natural populations is more time- and cost-effective, especially for woody perennial crops that have long life cycles and/or that require large planting areas. In oil palm, QTL mapping has been used to resolve the genetic bases of several complex traits, including yield components (Rance et al., 2001; Billotte et al., 2010; Jeennor and Volkaert, 2014; Tisne et al., 2015), fatty acid composition (Singh et al., 2009; Montoya et al., 2013), sex ratio (Ukoskit et al., 2014), and embryogenesis (Ting et al., 2013). Unfortunately, the contribution of QTL mapping studies to marker-assisted breeding outcomes has been less than expected. Although association mapping has been validated to be a reliable method for identifying trait-associated markers for marker-assisted selection (MAS), this method has rarely been applied to oil palm.

In this study, we used genome-wide association analysis (GWAS) analysis to identify SNP markers associated with fatty acid contents and identified candidate genes adjacent to these SNP markers based on our previous characterization of the genes involved in lipid metabolism pathways in oil palm (Xiao et al., 2019). We then analyzed the expression patterns of these candidate genes in different tissues. To validate the role of top candidate genes in regulating palmitic oil content, we transferred these genes into *A. thaliana* and over-expressed them. Our results provide a comprehensive understanding of fatty acid content in oil palm, and the SNPs and candidate genes detected will facilitate breeding for fatty acid content in oil palm.

## Materials and Methods

### Extraction and Measurement of Fatty Acid Contents in Oil Palm Mesocarp Tissues

Three fruits per oil palm individual (three biological replicates) were harvested, and fatty acid extraction and analyses for each mesocarp tissue were performed in triplicate (three different extractions as technical replicates). Approximately 60 mg of mesocarp (or 5 mg Arabidopsis seed) was used for extracting fatty acid according to methods described by Li-Beisson et al. (2013). Fatty acid composition was subsequently examined and measured using gas chromatography (Agilent DB-23, 30 m × 250). The heating procedure was as follows: the initial temperature was 180°C, followed by a temperature increase to 220°C at a rate of 10°C increase per minute. The gas pressure was 17.392 psi. The nine values (three biological replicates × three technical replicates) obtained per oil palm individual were averaged for subsequent association mapping. The contents of decanoic acid (C10:0), lauric acid (12:0), myristic acid (C14:0), tripalmitelaidin acid (16:1), palmitic acid (16:0), stearic acid (18:0), oleic acid (18:1), linoleic acid (18:2), and oil were determined with reference to an internal standard. The calculation formula was as follows: 1) total fatty acid = (total area of all measure fatty acid peaks × quantity of heptadecanoic acid-methyl ester)/(peak area of heptadecanoic acid-methyl ester × quantity of quantity of the sample); 2) relative fatty acid percentage = peak area of a specific fatty acid/total area of all measured fatty acid peaks. The peak area was calculated using the Agilent software.

### SNP Genotyping

A total of 1,261,501 reliable SNPs markers (minor allele frequency > 0.05 and integrity > 0.8) were previously identified based on SLAF-seq (specific locus amplified fragment sequencing) by an Illumina HiseqTM 2500 in a diversity panel of 200 oil palm individuals (Xia et al., 2019). The SLAF tag sequences were mapped to the African oil palm genome (Singh et al., 2013, the assembled oil palm genome of Version 5) using a mapping software: the Burrows-Wheeler alignment tool (Li and Drubin, 2009).

### Population Structure

Bayesian clustering was applied to analyze the population structure of 200 oil palm individuals using the software STRUCTURE (Pritchard et al., 2000). Based on the same set of SNPs, the number of subgroups (*K*) was predicted from 1 to 10, and the number of ancestors was determined according to the position of the minimum value, with an error rate obtained from 5-fold cross-validation. Maximum likelihood estimates for the ancestry proportion from each K subgroup for each accession were calculated. Five subgroups were indicated based on cross-validation errors (Xia et al., 2019).

### Association Mapping

To reduce spurious associations between alleles and phenotypic variation, mixed linear models (MLM) were used. Fixed effects were calculated using a Q (population) value matrix, and random effects were computed using a K (kinship) matrix. The Q+K value matrix was added to the MLM model. The Q matrix was obtained using STRUCTURE software (Pritchard et al., 2000), and the K matrix (genetic relationships among the 200 oil palm individuals) was obtained using SPAGeDi software (Hardy and Vekemans, 2002). Genome-wide association was performed using Tassel 5.0 (Bradbury et al., 2007). P-values for associations between SNP markers and fatty acid content were computed using the following formula:

y=Xβ+Sα+Qv+Zu+e

where *Xβ* represents the fixed effects other than the SNP under testing and the population structure; y is a vector of phenotypic observation; α is a vector of SNP effects; v is a vector of population effects; u is a vector of polygene backgroup effects; e is a vector of residual effects; Q is a matrix from backgroup effects relating y to v; and X, S, and Z are incidence matrices of 1s and 0s relating y to ß, α, and u, respectively (Yu et al., 2006). Quantile–quantile plots (Q–Q plots) were drawn using the “ggplot2” software R package (Ginestet, 2011), and the Manhattan plots was drawn using the “qqman” software package (Turner et al., 2014). A total of 237,714 SNP markers, which had no missing data in the 200 oil palm individuals, were selected for association analysis. The threshold value (P < 4.2e^−7^, −log_10_ *^P^* approximately equal to 6.3) was set for detecting reliable trait-associated markers at a cut-off FDR (false discovery rate) of 0.1 (Benjamini and Hochberg, 1995). Meanwhile, when FDR was set as 0.01, the threshold value of P value and −log_10_ *^P^* are 4.2e^−8^ and 7.3, respectively.

### Prediction of Candidate Genes Associated With Fatty Acid Content

All genes in LD blocks (*r* ^2^ > 0.6) containing SNPs that were significantly associated with traits were identified for further candidate gene selection (Raman et al., 2015). The LD blocks present in the 200 oil palm individuals were estimated using the software Haploview v4.2. The number and size of the LD blocks on each chromosome were calculated according to previously established methods (Barrett et al., 2005). If SNP markers significantly associated with traits were located outside LD blocks, candidate genes were selected for further analysis following the criteria used by Zhou et al. (2017). Amino sequences of these selected genes were aligned using BLAST against the *Arabidopsis* protein database to predict the potential function of candidate genes. All candidate genes were selected based on gene ontology (GO) terms related to fatty acid biosynthesis and metabolism, and all transcription factors were selected based on Clusters of Orthologous Group of proteins (COG) within SNP-tagged genome regions.

### Transcriptome Data Downloaded and RPKM Calculation

We downloaded transcriptomic raw read data from the SRA (Short Read Archive) database, including SRR851069 (mesocarp 10 weeks after anthesis), SRR851067 (mesocarp 15 weeks after anthesis), SRR190699 (mesocarp 17 weeks after anthesis), SRR190700 (mesocarp 19 weeks after anthesis), SRR190701 (mesocarp 21 weeks after anthesis), SRR190702 (mesocarp 23 weeks after anthesis), SRR851070 (kernel 10 weeks after anthesis), SRR851068 (kernel 15 weeks after anthesis), SRR190703 (leaf), SRR851071 (root), SRR851103 (shoot), SRR851101 (female flower), and SRR851099 (pollen). RPKM (reads per kb per million reads) were used to calculate gene expression levels using the following formula (Mortazavi et al., 2008):

$$\mathrm{RPKM}=\frac{10^{6}C}{\mathrm{NL}/10^{3}}$$

where C is the number of reads that aligned exclusively with one expressed sequence, N is the total number of reads that aligned with all expressed sequences, and L is the number of bases in the corresponding coding sequence.

### Vector Construction and Transformation for Over-Expression of *EgFatB*

Among the candidate genes, acyl-ACP thioesterase B genes *EgFatB1* and *EgFatB2* were significantly associated with high palmitic acid contents in the mesocarp. Primers used for *EgFatB* gene cloning were designed using the Snapgene Viewer software (**Table 1**). Polymerase chain reaction (PCR) amplifications were performed in 50 µl reaction mixtures containing a 500 ng cDNA sample from the oil palm mesocarp, 1 × PCR buffer, 2 mM MgCl_2_, 5 U of TaqDNA polymerase (TaKaRa, China), 0.5 μM of each primer, and 0.2 mM dNTP mix. The PCR program included denaturation at 98°C for 30 s, followed by 30 cycles at 98°C for 10 s, 55°C for 30 s, and 72°C for 60 s, and a final extension at 72°C for 5 min. The PCR products were electrophoretically visualized on a 1% agarose gel and recombined into the *pBinGlyRed3* vector (containing DsRed as a reporter gene and 35S as a promoter), which was then digested with EcoR I and XhoI. The constructed vectors were transformed into competent *Escherichia coli* cells (line Dh5l), and inserts were validated by sequencing. A plasmid with the correct sequence insert was transformed into *Agrobacterium* *GV3101* and then transformed into *A. thaliana* using an *Agrobacterium*-mediated *in planta* transformation approach (Clough et al., 1998). The detailed protocols were as follows: the transformed *Agrobacterium* strain was streaked on solid LB medium containing Gent and Kana antibiotic overnight (28°C) and then a single validated colony was propagated in the liquid LB medium (28°C, 250 r/min) for 16 h. The turbid *Agrobacterium* liquid (300 ml) was then centrifuged at a rotation speed of 5000 r/min for 20 min, and the precipitation was suspended by a solution with 5% sucrose and 0.001% SilvetL-77 after removing the liquid LB medium. *Arabidopsis* inflorescences at the 3–4 day post-bolting stage were immersed in the *Agrobacterium* suspension and covered with plastic wrap for 16 h in the dark. After 1 week, these steps were repeated to increase transformation efficiency. Positive transgenic *Arabidopsis* seeds were confirmed by detection of red autofluorescence and by PCR validation of targeted genes. Control and transgenic *Arabidopsis* plants were grown with a 16 h light/8 h dark photoperiod at 25°C. Fatty acid composition of transformed *Arabidopsis* plants was examined with the same protocol as was used for oil palm mesocarp tissue.

**Table 1:** Primer sequences used to amplify the full CDS sequence of FatB1 and FatB2 in oil palm (*E. guineensis*).

	Forward primer	Reverse primer
FatB1	CC**GGAATT**CAGTGTCTCCATATCCCCATC	CCG**CTCGAG**TCAGTATTTCAAACGCAACA

Nucleotides in bold represent restriction enzyme sites for EcoR I and Xho I.

## Results

### Fatty Acid Composition in the Oil Palm Population

**Figure 2:**
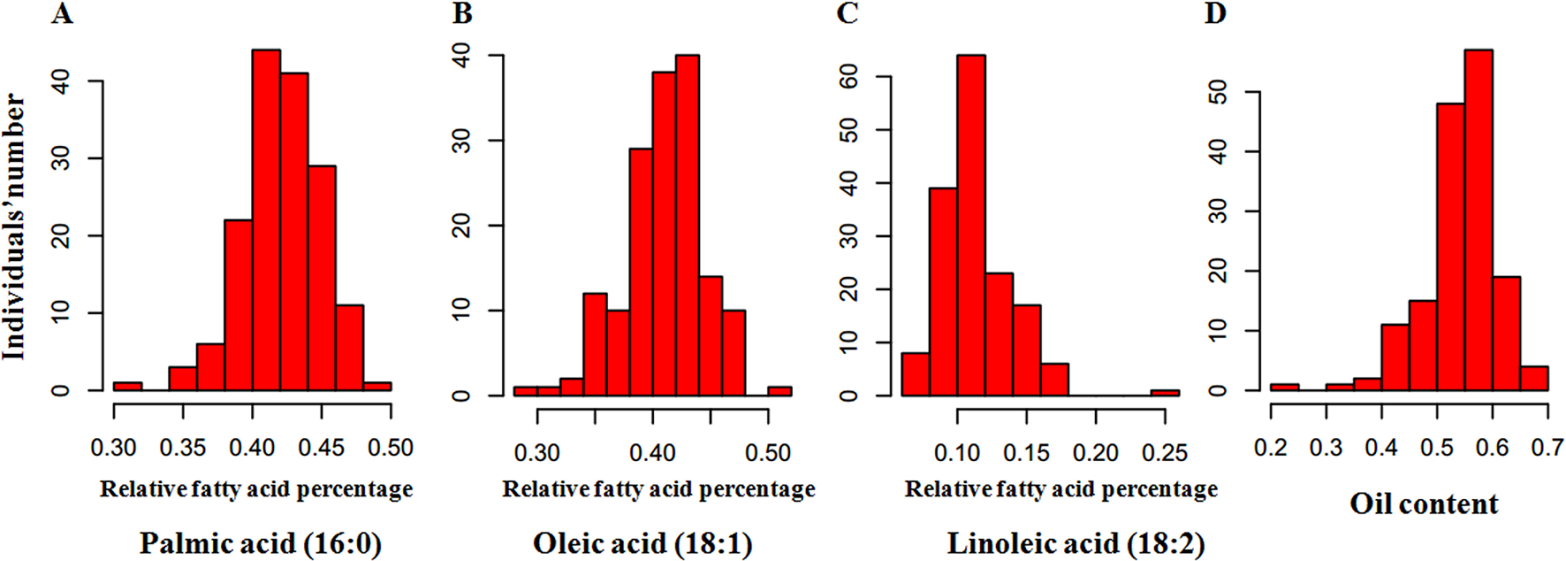
Frequency distribution of relative fatty acid percentages in the mesocarp of 200 oil palm individuals, including palmitic acid (16:0) **(A)**, oleic acid (18:1) **(B)**, linoleic acid (18:2) **(C)**, and oil **(D)**. The x-axis represents the trait value, and the y-axis represents the number of oil palm individuals.

To perform an association analysis between SNP markers and relative fatty acid percentage, we first determined the relative fatty acid percentage of each of the 200 oil palm individuals from different geographical origins (**Figure 2**). Among the 200 oil palm individuals, relative palmitic acid percentage ranged from 31.3 to 48.8% with an average of 42.0%, oleic acid ranged from 31.3 to 50.1%, linoleic acid ranged from 7.1 to 18.5%, and total oil content ranged from 29.8 to 70.3%. Relative palmitic acid percentage (C16:0) was negatively associated with stearic acid (*r* = −0.627; P < 0.001) and relative oleic acid content (*r* = −0.657; P < 0.001). Relative oleic acid percentage was also negatively correlated with linoleic acid content (*r* = −0.689; P < 0.001).

### Genome-Wide Associations Between SNPs and Fatty Acid Content

**Figure 3:**
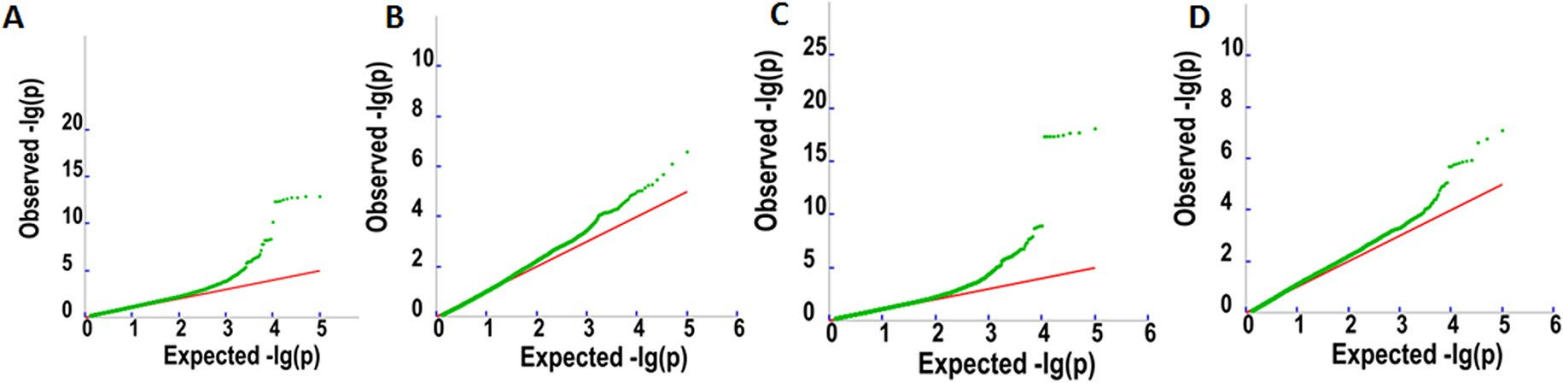
Q–Q plots for oil content **(A)**, palmitic acid content (C16:0) **(B)**, linoleic acid content (18:2) **(C)**, and oleic acid content (18:1) **(D)**.

**Figure 4:**
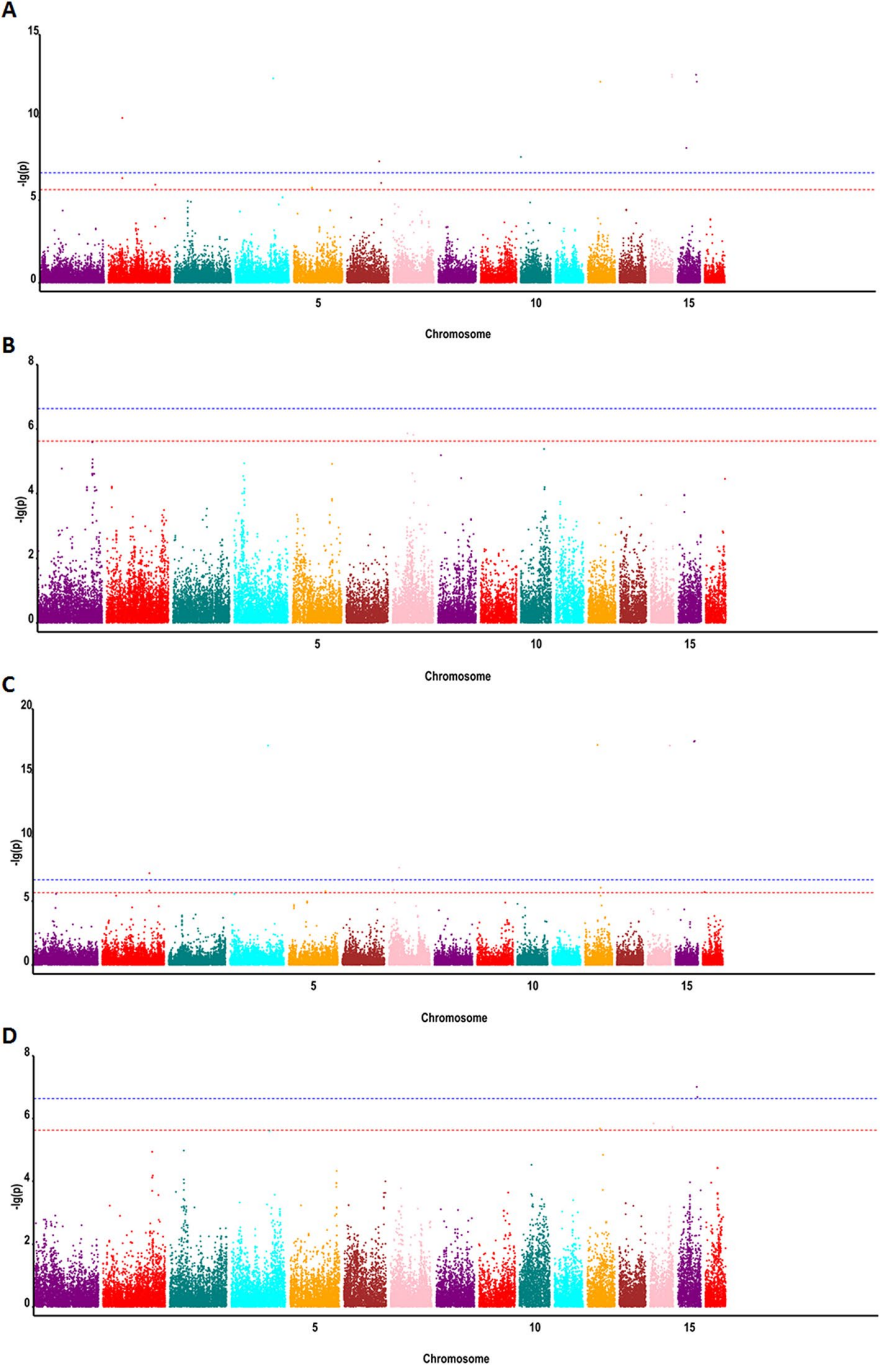
Genome-wide associations between SNP markers and fatty acid contents in oil palm (*E. guineensis*) using MLM: **(A)** Manhattan plot for oil content, **(B)** Manhattan plot for palmitic acid content (C16:0), **(C)** Manhattan plot for linoleic acid content (18:2), and **(D)** Manhattan plot for oleic acid content (18:1). Red (6.3) and blue (7.3) lines indicate P-value cutoffs FDR of 0.1 and 0.01, respectively.

To detect associations between allele variation and fatty acid content, we used MLM for GWAS; this controlled for the presence of population structure in the analyzed oil palm population. Meanwhile, five subgroups were previously indicated based on cross-validation errors (Xia et al., 2019) (see **Supplementary Table 1** for Q values). The MLM analysis revealed 62 SNP markers that were significantly associated (P < 4.2e^−7^) with fatty acid content, including palmitic acid content (32 SNPs), oleic acid content (4 SNPs), linoleic acid content (1 SNP), and total oil content (25 SNPs) (**Figures 3** and **4**).

### Identification of Candidate Genes Related to Fatty Acid Composition in *E. guineensis*

Of the 62 SNP markers significantly associated with fatty acid content, 6 were located in different LD blocks (*r* ^2^ > 0.6), while the other SNP markers were not located in defined LD blocks. A total of 223 candidate genes were identified in the flanking regions of SNP markers that were significantly associated with different fatty acid contents (**Supplementary Table 2**). Based on GO annotation results, one candidate gene was involved in fatty acid biosynthesis: an acyl-ACP thioesterase B gene (*FatB*, involved in the termination of the fatty acid chain).

### Candidate Gene Expression in Different Oil Palm Tissues

**Figure 5:**
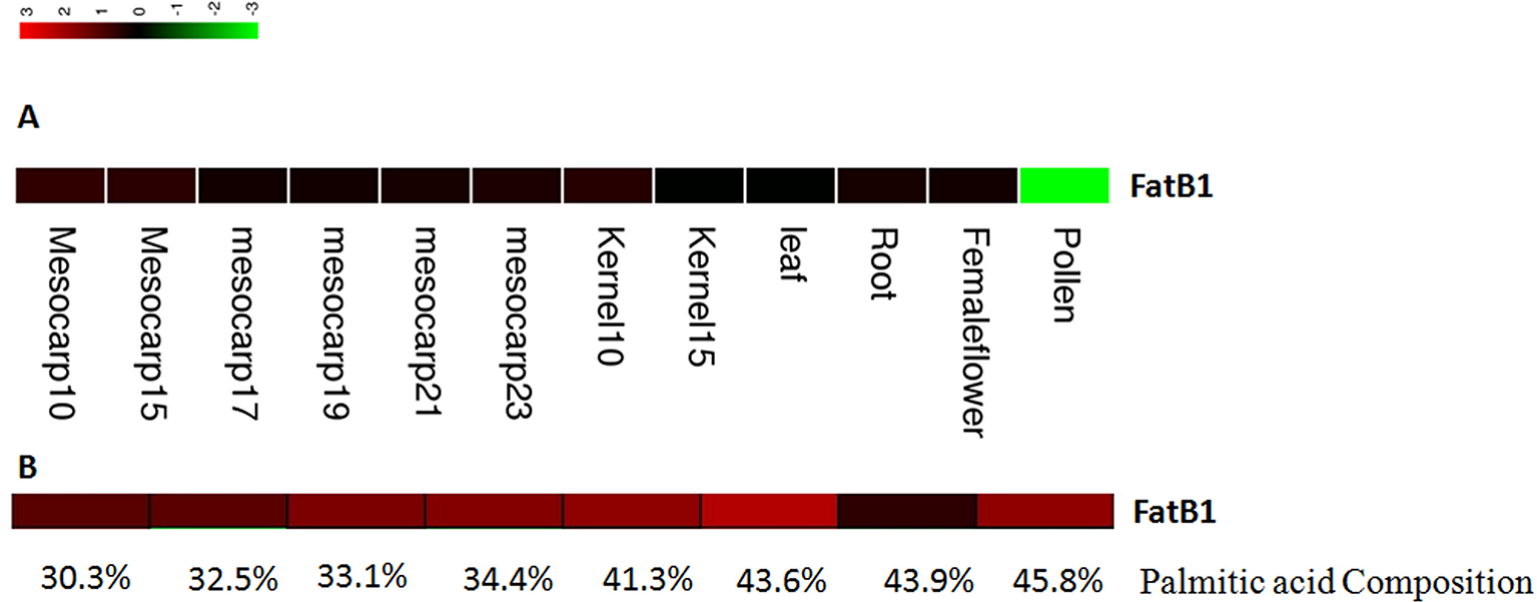
Expression of *EgFatB2* in different tissues **(A)** and in individuals with differing palmitic acid content **(B)**.

The expression levels of candidate genes in fatty acid biosynthesis and metabolism pathways in different tissues and at different mesocarp developmental stages were estimated using RPKM values based on the method of Mortazavi et al. (2008). One candidate gene involved in fatty acid biosynthesis and metabolism was identified based on GO annotation results, and this gene showed higher expression levels in the mesocarp tissues than in other tissue types (**Figure 5A**). The RPKM values for this gene were 41.8 and 37.9 at 10 and 15 weeks post-anthesis.

One *FatB* gene was detected as near to one significant SNP (EgChr7, 1731788). *FatB* genes have been reported to catalyze the termination of saturated fatty acyl-ACP. Interestingly, the expression level of *EgFatB1* was generally higher in the mesocarp than in the kernel, especially at 10 and 15 weeks post-anthesis.

### Candidate Gene Expression Profiles in Different Oil Palm Individuals

In order to analyze the expression levels of candidate genes in different oil palm individuals with different fatty acid contents, we performed RNA-Seq on eight oil palm individuals. Four oil palm individuals were chosen on the basis of higher palmitic acid content (45.8%, 43.6%, 43.9%, and 41.3%) and showed *FatB* gene RPKM values of 52.8, 133.6, 3.45, and 49.9, respectively (**Figure 5B**). Four additional oil palm individuals were chosen on the basis of lower palmitic acid content (37.1%, 32.5%, 33.1%, and 30.3%) and showed *FatB* gene RPKM values of 37.1, 12.2, 29.9, and 11.6, respectively. The average expression level of *FatB* in oil palm individuals with higher palmitic acid content was two-fold higher than that of individuals with lower palmitic acid content.

### Functional Validation of *Egfatb1*

**Figure 6:**
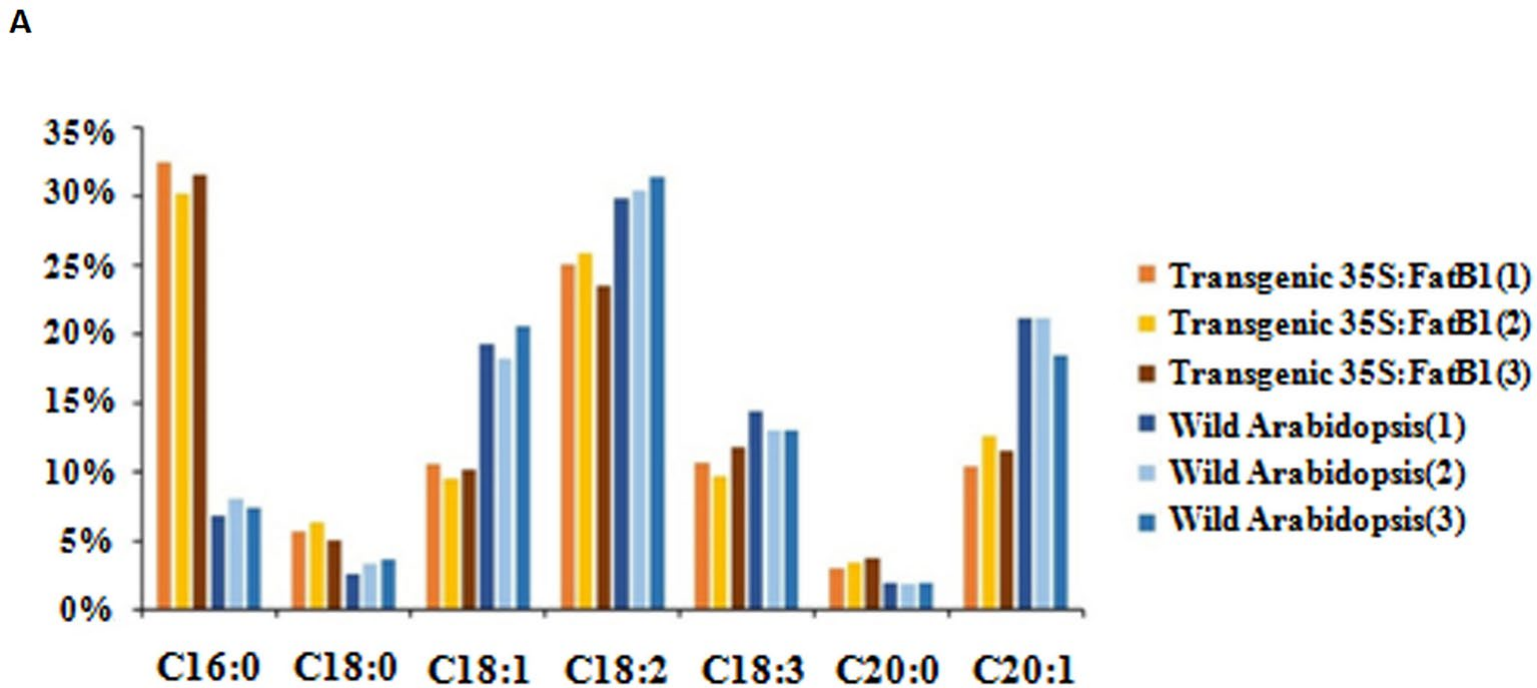
Fatty acid composition in transgenic *35S:EgFatB1* and wild-type *A. thaliana.* The X axis represented fatty acid content and the Y axis represented the fatty acid components..

The full length coding sequence of *EgFatB1* was cloned and inserted into the pBinGlyRed3 vector. Genes inserted into this vector are promoted by a 35S promoter and in the same frame as the red autofluorescence reporter gene (DsRed3). The *EgFatB1* over-expression plasmid was transformed into *Arabidopsis*. Positive transformants were screened in the T1 generation by detection of red autofluorescence in the seeds and by PCR validation. Positive transgenic plants in the T2 generation were investigated for fatty acid contents. Transgenic plants with over-expressed *EgFatB1* increased the palmitic acid and stearic acid content but decreased the oleic acid and linoleic acid content in the seeds (**Figure 6**).

## Discussion

Oil palm (*E. guineensis*) is an important crop for human nutrition, with tens of millions of tonnes of palm oil consumed every year. Although breeding could potentially improve the nutritional value of palm oil for human consumption (e.g. by lowering the saturated fat content), breeding is hindered by the lack of genetic and genomic resources available for this crop. Using SLAF-seq technology, we genotyped a diversity panel of 200 oil palm individuals from four countries to obtain 1.2 million genome-wide SNPs (Xia et al., 2019). We associated these SNPs with fatty acid content and identified dozens of candidate genes involved in fatty acid biosynthesis and metabolism pathways. One gene, *EgFatB1*, which was highly and specifically expressed in the mesocarp, was validated using *Arabidopsis* transformation: over-expression of *EgFatB1* resulted in increased saturated fat content in *Arabidopsis* seeds.

Oil palm (*E. guineensis*) originated from West and Southwest Africa, especially in the area between Angola and Gambia. Oil palm is well-known for its highest oil yield per unit area. Palm oil is an edible vegetable oil and derived from the mesocarp (reddish pulp) of the oil palm fruit. Along with coconut oil, palm oil is semi-solid at room temperature due to a high content of saturated fatty acid, especially palmitic acid (C16:0), which accounts for approximately 50% of palm oil fatty acid content. However, how fatty acid composition varies in different oil palm germplasm is unknown. In this study, we investigated the variation of fatty acid composition among 200 oil palm individuals. A large variation in fatty acid composition was detected between these oil palm individuals. Our study identified useful oil palm germplasm for improving fatty acid composition, especially for decreasing palmitic acid content (lowest value: 31.3%) and increasing oleic acid content (highest value: 50.1%).

GWAS was firstly used as a powerful method to identify a broad range of complex diseases in human populations (Burton et al., 2007). Subsequently, with the completion and availability of additional genome sequences, this approach was used in flowering plants, including rice (Huang et al., 2011), foxtail millet (Jia et al., 2013), maize (Li et al., 2013), and *Arabidopsis* (Atwell et al., 2010). There are, however, some limiting factors in association analysis, such as insufficient marker density and population structure effects (Cappa et al., 2013). In a previous study, SLAF technology was used to develop a large number of SNP markers and to evaluate potential population structure, thus addressing these limiting factors and providing useful markers for MAS in oil palm. Palm oil accounts for 25% of vegetable oil traded worldwide annually and uses 5% of the global oil planting area. However, breeding progress in this tropical species is still slow, mostly due to the long life cycles of individual plants. Currently, only five to six generations of phenotype-based selection have been completed since the 1920s and 1930s (Hartley, 1967).

MAS has major potential to speed up the breeding of oil palm cultivars and reduce the length of conventional breeding cycles. Identifying trait-associated markers is a prerequisite for MAS. In the last decades, molecular markers have been used to identify QTL associated with important agronomic traits. Most marker development is based on conventional methods that result in modest density, including restriction fragment length polymorphism (RFLP) (Mayes et al., 1997), amplified fragments length polymorphism (AFLPs) (Singh et al., 2009), and simple sequence repeat (SSR) marker types (Billotte et al., 2010). To increase marker density, SNP markers were developed in oil palm. These high density SNP markers were successfully used in our study to identify molecular markers associated with important agronomic traits.

In oil palm, the first genetic linage map was constructed using RFLP markers (Rance et al., 2001). QTL mapping was subsequently used to decipher the genetic bases of yield components (Rance et al., 2001; Billotte et al., 2010), fatty acid composition (Singh et al., 2009; Montoya et al., 2013), and sex ratio (Ukoskit et al., 2014), among other traits. These QTL mapping experiments involved populations produced from one to four parents. Montoya et al. (2013) reported 19 QTLs associated with fatty acid composition in palm oil using a backcross population of *Elaeis oleifera* with *E. guineensis*. Acyl-ACP thioesterase A and Stearoyl-ACP desaturase genes were found located in these QTL intervals. However, the confidence interval of these QTLs ranged from 6 to 30 cM, or approximately 6–30 Mbp of physical genome sequence in oil palm (Montoya et al., 2013). Teh et al. (2016) identified three key loci for high mesocarp oil content in oil palm, which were located on Chr5, Chr9, and Chr11. In our study, we identified 62 SNP markers (P < 4.2e^−7^) significantly associated with fatty acid content on Chr3, Chr5, and Chr11. Meanwhile, we also identified SNP markers associated with fatty acid on Chr1, Chr6, Chr10, Chr12, and Chr13. The *EgFatB1* gene was identified on Chr6 and validated to be associated with variation in palmitic acid content.

In our study, some SNP markers were associated with variation in fatty acid content, which is useful for selecting low palmitic acid genotypes in oil palm breeding and subsequently improving the nutritional value of palm oil. Moreover, there have been some previous examples where fatty acid composition in different crops has been improved using various biotechnology and mutation approaches. In rapeseed, the relative oleic acid content was increased to 71% by EMS and ^60^Co mutagenesis (Rucker and Robbelen, 1995). In soybean, oleic acid content increased to 85% in a *fad2-1* mutant (Wagner et al., 2011). Meanwhile, in peanut, Norden et al. (1987) bred a high oleic acid cultivar (F435) from a natural mutant (Norden et al., 1987). Hence, artificial mutagenesis in oil palm could produce germplasm with significant divergence in fatty acid composition. In the present study, *FatB* was validated as a major effect gene for relative palmitic acid content, allowing us to screen for low palmitic acid content in oil palm by selecting for *FatB* mutations. The availability of molecular markers associated with agronomic traits will allow breeders to rapidly identify target traits in seedlings, thus accelerating selection and breeding schemes. Meanwhile, molecular markers associated with agronomic traits can also be used to detect the introgression of the trait of interest into elite varieties from more diverse germplasm.

## Data Availability Statement

The raw SLAF-seq data for the 200 oil palm individuals has been deposited into the European Nucleotide Archive (http://www.ebi.ac.uk/ena). The bioproject number is PRJEB26466.

## Author Contributions

YX, WX, and CZ participated in the design of the study. YX and WX performed the statistical analysis and drafted the manuscript. JW and YD were involved in analysis of the added transcriptome datas. AM critically revised the manuscript. TL and WZ did the major experimental work including the extraction and measurement of oil content and relative fatty acid contents. WT, YD, DH, and XH contributed to and advised on the statistical analysis. All authors read and approved the final manuscript.

## Funding

This work was supported by the Scientific and Technological Cooperation Projects of Hainan province (No. KJHZ2015-06). AM is funded by DFG Emmy Noether grant MA6473/1-1.

## Conflict of Interest

The authors declare that the research was conducted in the absence of any commercial or financial relationships that could be construed as a potential conflict of interest.
